# Central Granular Cell Odontogenic Tumor of the Mandible: An Uncommon Presentation

**DOI:** 10.7759/cureus.49914

**Published:** 2023-12-04

**Authors:** Varun Rastogi, Siddharth Gupta, Nitin Sangwan, Nisha Maddheshiya, Karthikeyan Ramalingam

**Affiliations:** 1 Department of Oral Pathology, Universal College of Medical Sciences and Teaching Hospital, Bhairahawa, NPL; 2 Oral Medicine and Radiology, Universal College of Medical Sciences, Bhairahawa, NPL; 3 Periodontics, Universal College of Medical Sciences, Bhairahawa, NPL; 4 Oral Medicine and Radiology, Faculty of Medical Sciences, Institute of Medical Sciences, Banaras Hindu University, Varanasi, IND; 5 Oral Pathology and Microbiology, Saveetha Dental College and Hospitals, Saveetha Institute of Medical and Technical Sciences, Saveetha University, Chennai, IND

**Keywords:** immunohistochemistry, male, intraosseous, mandible, granular cell tumor, granular cell, central, odontogenic tumor

## Abstract

Central granular cell odontogenic tumor (CGCOT) is a rare, benign odontogenic tumor resulting from the jaw bone, especially the mandible or maxilla. It affects women of middle age and usually occurs as a painless swelling of the mandibular premolar-molar area. CGCOT is characterized by the presence of granular cells, which are large, eosinophilic, granular-looking cells found in the tumor tissue.

We report an unusual CGCOT in a 38-year-old male patient's mandibular region. We also describe the clinical, radiological, and pathological characteristics along with the immunohistochemical investigation of the tumor.

## Introduction

Central granular cell odontogenic tumor (CGCOT) is a rare and intriguing entity within the spectrum of odontogenic tumors. CGCOT is characterized by the presence of eosinophilic granules within the cell, and it is considered a benign neoplasm originating from odontogenic mesenchyme [1]. Although the latest WHO classification of odontogenic tumors does not officially recognize CGCOT, many experts in the field consider it to be a distinct and separate tumor entity [2]. There are 51 reported cases in the literature to the best of our knowledge, to date.

CGCOT represents 0.3% of odontogenic tumors and primarily affects the jawbones, with the mandible being the more common site, although cases involving the maxilla have also been reported. The tumor is usually a painless and slow-growing swelling of the jaw in the premolar-molar region and is often found accidentally during a routine dental radiographic examination [3,4]. While it is generally benign, its rarity and shared features with other odontogenic lesions often make it challenging to diagnose definitively. The paucity of this tumor in middle-aged men and the specific diagnostic findings, including the immunohistochemical markers used, make this case worthy of documentation. By sharing this case, we aim to contribute to the limited literature on CGCOT and enhance our understanding of its clinical, radiographic, and histopathological characteristics, ultimately facilitating more accurate diagnosis and management of similar cases in the future.

## Case presentation

A 38-year-old male patient reported that he had pain in the lower right back teeth area for the past four months. His past medical, surgical, and dental history was non-contributory. No noticeable swelling was observed externally or within the mouth during the clinical examination. To assess the condition, an orthopantomograph (OPG) was recommended.

**Figure 1:**
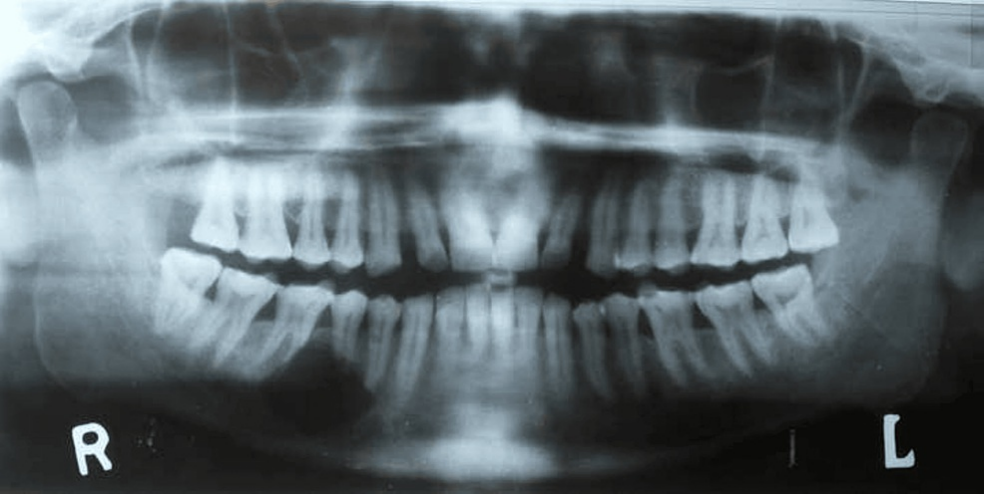
Orthopantomogram Orthopantomogram demonstrating well-defined radiolucency with root resorption in relation to the 44 to 46 region of the right mandible

The OPG showed a clearly defined and unilocular radiolucency near the 44 to 46 teeth, with evidence of root resorption occurring at tooth 45 and the mesial root of tooth 46 (Figure 1).

**Figure 2:**
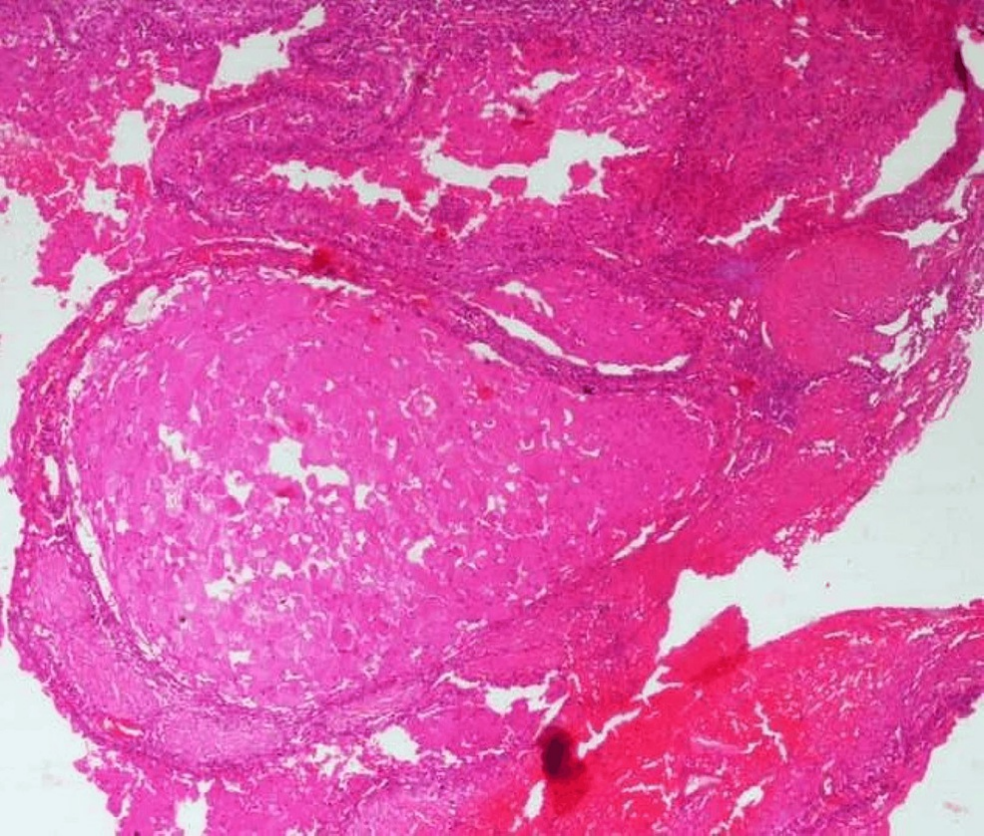
Photomicrograph Photomicrograph showing islands of granular cells surrounded by fibrous connective tissue stroma and areas of hemorrhage (H & E stain, 10x view)

An aspiration test yielded negative results. Consequently, an incisional biopsy was performed under local anesthesia to obtain a tissue sample that was fixed with 10% formalin for subsequent histopathological examination. Hematoxylin-and-eosin-stained soft tissue sections showed odontogenic epithelium islands surrounded by a thin fibrocellular connective tissue stroma and areas of hemorrhage (Figure 2).

**Figure 3:**
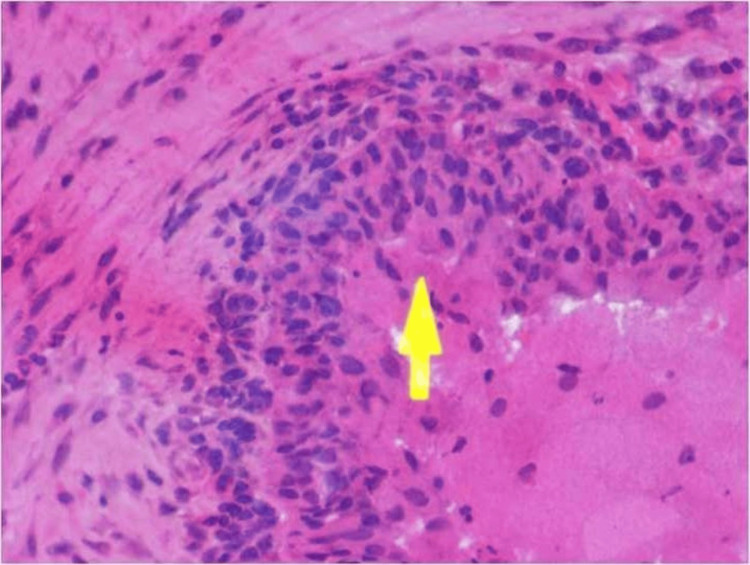
Photomicrograph Photomicrograph showing central cells with granular cytoplasm and an eccentrically placed nucleus (H & E stain, 40x view)

Epithelial islands showed large central granular cells with eccentrically placed nuclei and abundant eosinophilic granules within their cytoplasm (Figure 3).

**Figure 4:**
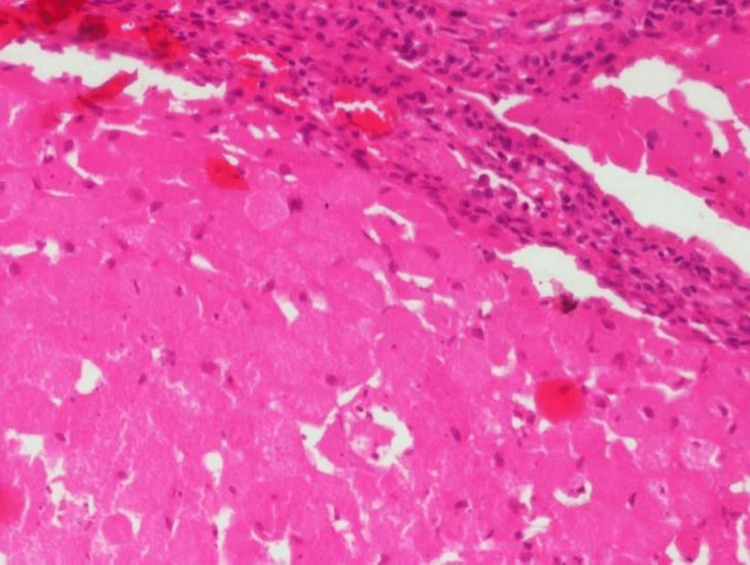
Photomicrograph Photomicrograph showing central cells with granular cytoplasm and eccentrically placed nucleus with cuboidal to low columnar peripheral cells (H & E stain, 40x view)

Peripheral cells were cuboidal to low columnar without any nuclear palisading and no reversal of polarity (Figure 4).

**Figure 5:**
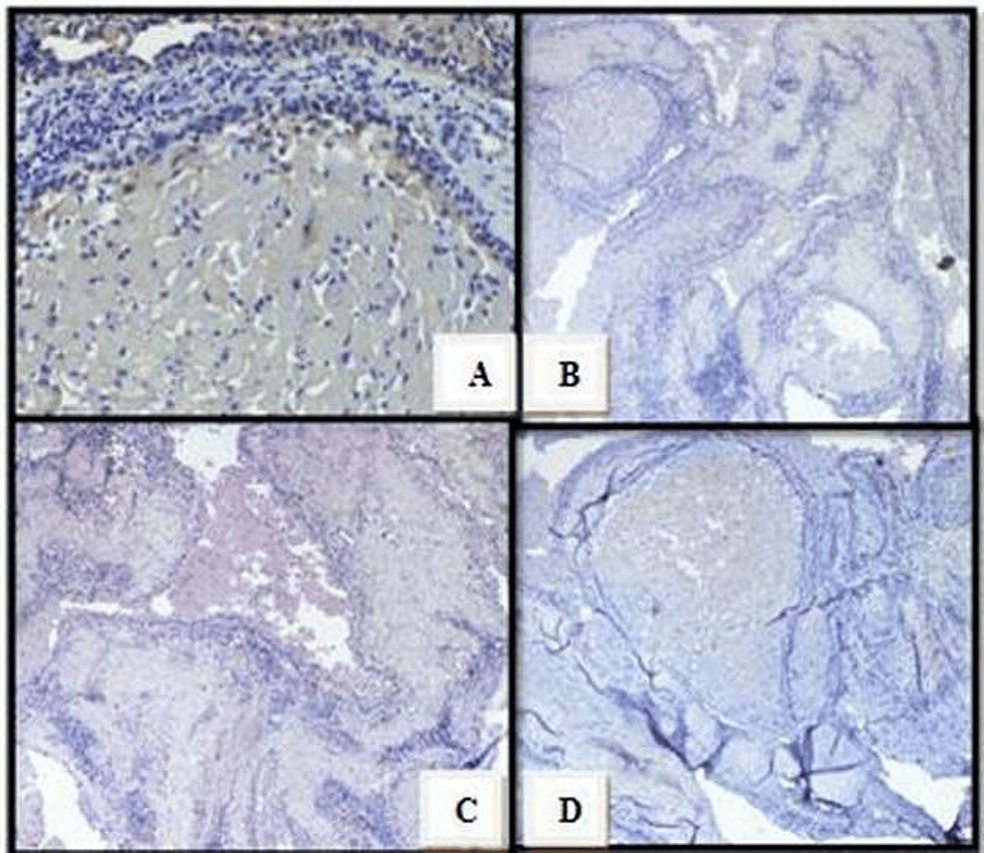
Photomicrograph Photomicrograph showing Pan-cytokeratin positivity (A), Calretinin negativity (B), S-100 negativity (C), and Neuron Specific Enolase (NSE) negativity (D) on immunohistochemical assessment

Immunohistochemical staining was performed with the Biogenex Immunohistochemical Kit (Biogenex, Fremont, CA, USA) as per manufacturer instructions for Pan-cytokeratin (Pan CK), Calretinin, S-100, and Neuron Specific Enolase (NSE). Pan CK (AE1/AE3) was positively stained in peripheral cells and negative in central granular cells. Calretinin, S-100, and NSE were also negative (Figure 5).

Correlating with the radiological features, histopathological findings, and immunohistochemical observations, the final diagnosis was given as CGCOT.

## Discussion

CGCOT has undergone several name changes and classifications over the years. Mashhadiabbas F reported the detailed history and first usage of the descriptive term 'central granular cell odontogenic fibroma' [1]. Tumors composed mainly of granular cells with ameloblastic and/or odontogenic characteristics are recognized as separate entities and called granular cell odontogenic tumors. However, it is significant to note that despite various names and classifications over the years, the 2017 WHO classification of odontogenic tumors did not include it as a separate category [5].

CGCOT is known to manifest across a broad age spectrum, with reported cases occurring between 16 to 77 years, and the average age of onset stands at 45.21 years. Most cases occur in individuals in their fifth to seventh decades of life. In terms of gender distribution, females have a predominance, with a ratio of 3.1 to 1 between females and males [6]. We present a case involving a 38-year-old male patient.

The duration can vary significantly, with reported cases spanning from 5 months to as long as 19 years [2]. Our patient reported a four-month duration. Some individuals may exhibit entirely asymptomatic lesions while others may present with a painless mass that causes localized expansion in the affected area [7]. It's noteworthy that there have been very few documented cases of GCOTs occurring in the gingiva [8]. Our patient presented with pain in the lower jaw.

In terms of demographics, this condition has been primarily reported in individuals of black and white ethnic backgrounds. However, Chien et al. reported the first case of CGCOT in people of Eastern or Oriental descent, suggesting that tumors can be present in different ethnic or racial groups [2]. These demographic and clinical characteristics provide valuable insights into the profile of CGCOT cases, aiding in the understanding and diagnosis of this rare odontogenic tumor.

Regarding anatomical distribution, CGCOT shows a prevalence of 72.02% in the mandible and 27.7% in the maxilla [6]. Our case was also involving the mandibular molar region. CGCOT is usually radiographically presented as a well-defined unilocular or multilocular radiolucency, and in some cases, focal areas of opacity may be observed [9]. Our case also presented a well-defined radiolucency in the mandibular region.

Histopathologically, the tumor is characterized by the presence of sheets and clusters of cells, which are round to polygonal and exhibit abundant, finely granular, eosinophilic cytoplasm. The nucleus is often eccentrically placed within the cell, and mitotic activity is usually absent [10]. The peripheral cells often appear as low columnar or cuboidal cells, and the tumor typically lacks the presence of stellate reticulum-like cells. Additionally, in some instances, dystrophic calcifications may be observed within the tumor. The granular cells found in CGCOT contain numerous eosinophilic granules, which can be identified through histopathological examination.

Meer et al.'s research suggests that CGCOT granular cells may have a mesenchymal origin, a finding supported by their positive staining for vimentin, and suggested that they may be derived from a histiocytic cell lineage [11]. This granular cell histiocytic differentiation was further supported by Gomes et al's research, which observed strong expression of CD68, a well-known histiocytic marker [12]. In addition, ultrastructural analysis of granular cells revealed minimal cytoplasmic organelles, but a large number of electron-dense lysosome-like particles within the cytoplasm [13]. Takeda et al proposed that the granular changes observed in CGCOT may indicate degenerative processes or aging rather than a neoplastic origin [14]. These findings contribute to the ongoing exploration of the origin and nature of granular cells in CGCOT.

However, immunohistochemical markers such as calretinin, AE1/AE3, S100, and neuron-specific enolase (NSEs) can help significantly in diagnosis by verifying the tumor's odontogenic origin [2]. Our case showed classic histopathological features along with positive expression of Pan-CK in the peripheral cells. The differential diagnosis of granular cell lesions within the oral cavity includes granular cell tumors of soft tissues, granular cell ameloblastoma, congenital epulis of the newborn, and peripheral granular cell odontogenic fibroma [6,7]. Granular cell tumors of soft tissues are strongly positive for S-100. Granular cell changes in ameloblastoma are positive for cytokeratin and negative for S-100. Congenital epulis is positive for NSE but negative for S-100 [15].

The majority of the reported cases have been successfully managed through enucleation and curettage. These procedures have proven to be effective in the removal of the lesions, and the recurrence rate is notably low. As a result, the prognosis for individuals with CGCOT is generally favorable [7,15]. Our patient was also referred for surgical enucleation at a higher center for further management.

## Conclusions

Central granular cell odontogenic tumor (CGCOT) is a rare but distinct odontogenic entity. Accurate diagnosis through clinical, radiographic, histopathological, and immunohistochemical evaluations is essential and emphasizes the importance of considering this rare tumor in the differential diagnosis of jaw lesions, even in atypical patient demographics. Despite its rarity, early diagnosis and effective treatment can result in a favorable prognosis. Further research and reporting are essential to expand our understanding of this intriguing odontogenic tumor. CGCOT is often underrepresented in the literature, making case reports like this valuable for expanding our understanding of the condition and improving patient care.
